# *Trichoderma* spp. Genes Involved in the Biocontrol Activity Against *Rhizoctonia solani*

**DOI:** 10.3389/fmicb.2022.884469

**Published:** 2022-05-25

**Authors:** Aqleem Abbas, Mustansar Mubeen, Hongxia Zheng, Muhammad Aamir Sohail, Qaiser Shakeel, Manoj Kumar Solanki, Yasir Iftikhar, Sagar Sharma, Brijendra Kumar Kashyap, Sarfaraz Hussain, Maria del Carmen Zuñiga Romano, Ernesto A. Moya-Elizondo, Lei Zhou

**Affiliations:** ^1^State Key Laboratory for Managing Biotic and Chemical Threats to the Quality and Safety of Agro-Products, Institute of Agro-Product Safety and Nutrition, Zhejiang Academy of Agricultural Sciences, Hangzhou, China; ^2^College of Plant Science and Technology, Huazhong Agricultural University, Wuhan, China; ^3^Department of Plant Pathology, College of Agriculture, University of Sargodha, Sargodha, Pakistan; ^4^Department of Plant Pathology, Faculty of Agriculture and Environment, The Islamia University of Bahawalpur, Bahawalpur, Pakistan; ^5^Plant Cytogenetics and Molecular Biology Group, Institute of Biology, Biotechnology and Environmental Protection, Faculty of Natural Sciences, University of Silesia in Katowice, Katowice, Poland; ^6^Department of Biotechnology Engineering, Institute of Engineering and Technology, Bundelkhand University, Jhansi, India; ^7^Institute of Food Science and Technology, Chinese Academy of Agricultural Sciences, Beijing, China; ^8^Fitosanidad-Fitopatología, Colegio de Postgraduados, Montecillo, Mexico; ^9^Department of Plant Production, Faculty of Agronomy, Universidad de Concepción, Chillán, Chile

**Keywords:** *Trichoderma* spp., genes, *R. solani*, antibiosis, competition, mycoparasitism, induced systemic resistance

## Abstract

*Rhizoctonia solani* is a pathogen that causes considerable harm to plants worldwide. In the absence of hosts, *R. solani* survives in the soil by forming sclerotia, and management methods, such as cultivar breeding, crop rotations, and fungicide sprays, are insufficient and/or inefficient in controlling *R. solani*. One of the most challenging problems facing agriculture in the twenty-first century besides with the impact of global warming. Environmentally friendly techniques of crop production and improved agricultural practices are essential for long-term food security. *Trichoderma* spp. could serve as an excellent example of a model fungus to enhance crop productivity in a sustainable way. Among biocontrol mechanisms, mycoparasitism, competition, and antibiosis are the fundamental mechanisms by which *Trichoderma* spp. defend against *R. solani*, thereby preventing or obstructing its proliferation. Additionally, *Trichoderma* spp. induce a mixed induced systemic resistance (ISR) or systemic acquired resistance (SAR) in plants against *R. solani*, known as *Trichoderma*-ISR. Stimulation of every biocontrol mechanism involves *Trichoderma* spp. genes responsible for encoding secondary metabolites, siderophores, signaling molecules, enzymes for cell wall degradation, and plant growth regulators. *Rhizoctonia solani* biological control through genes of *Trichoderma* spp. is summarized in this paper. It also gives information on the *Trichoderma*-ISR in plants against *R. solani*. Nonetheless, fast-paced current research on *Trichoderma* spp. is required to properly utilize their true potential against diseases caused by *R. solani*.

## Introduction

**Figure 1:**
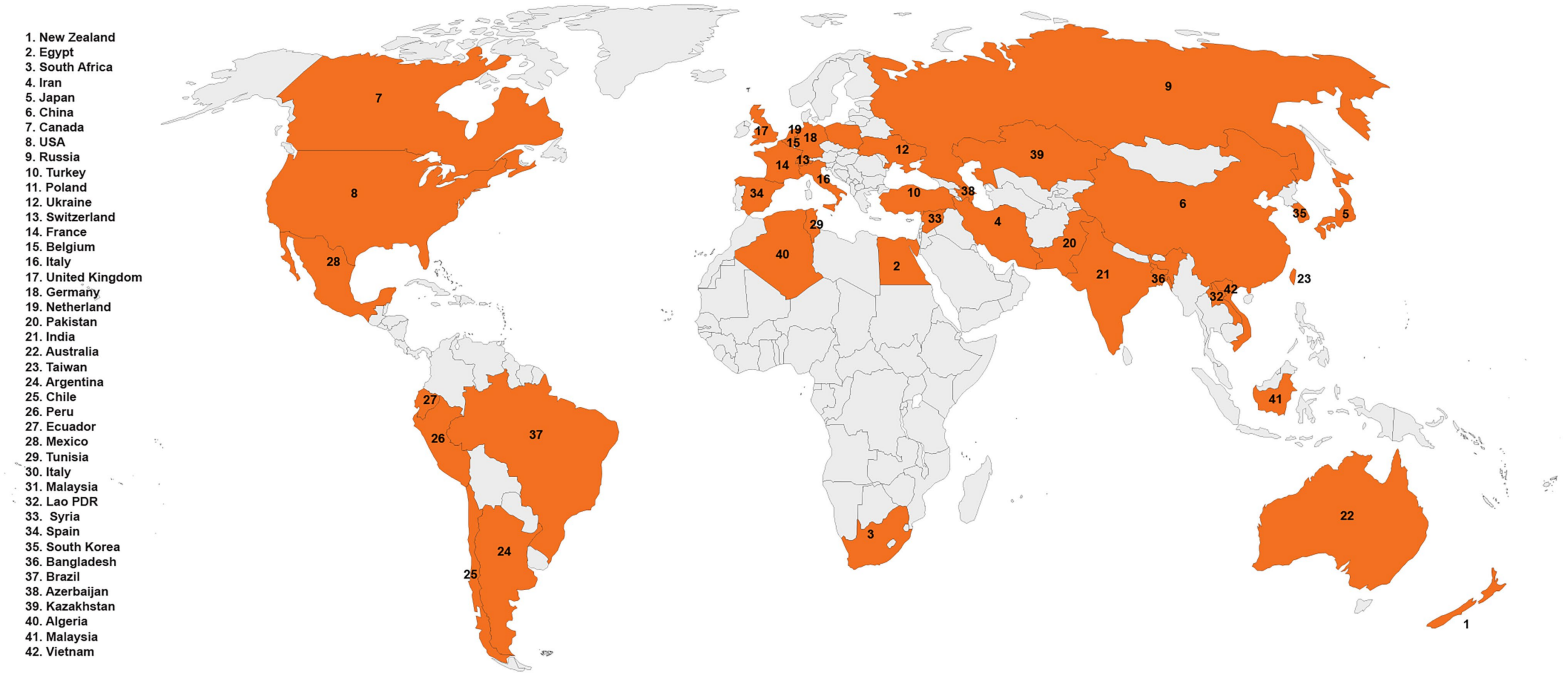
Geographical distribution of *Rhizoctonia solani.*

**Figure 2:**
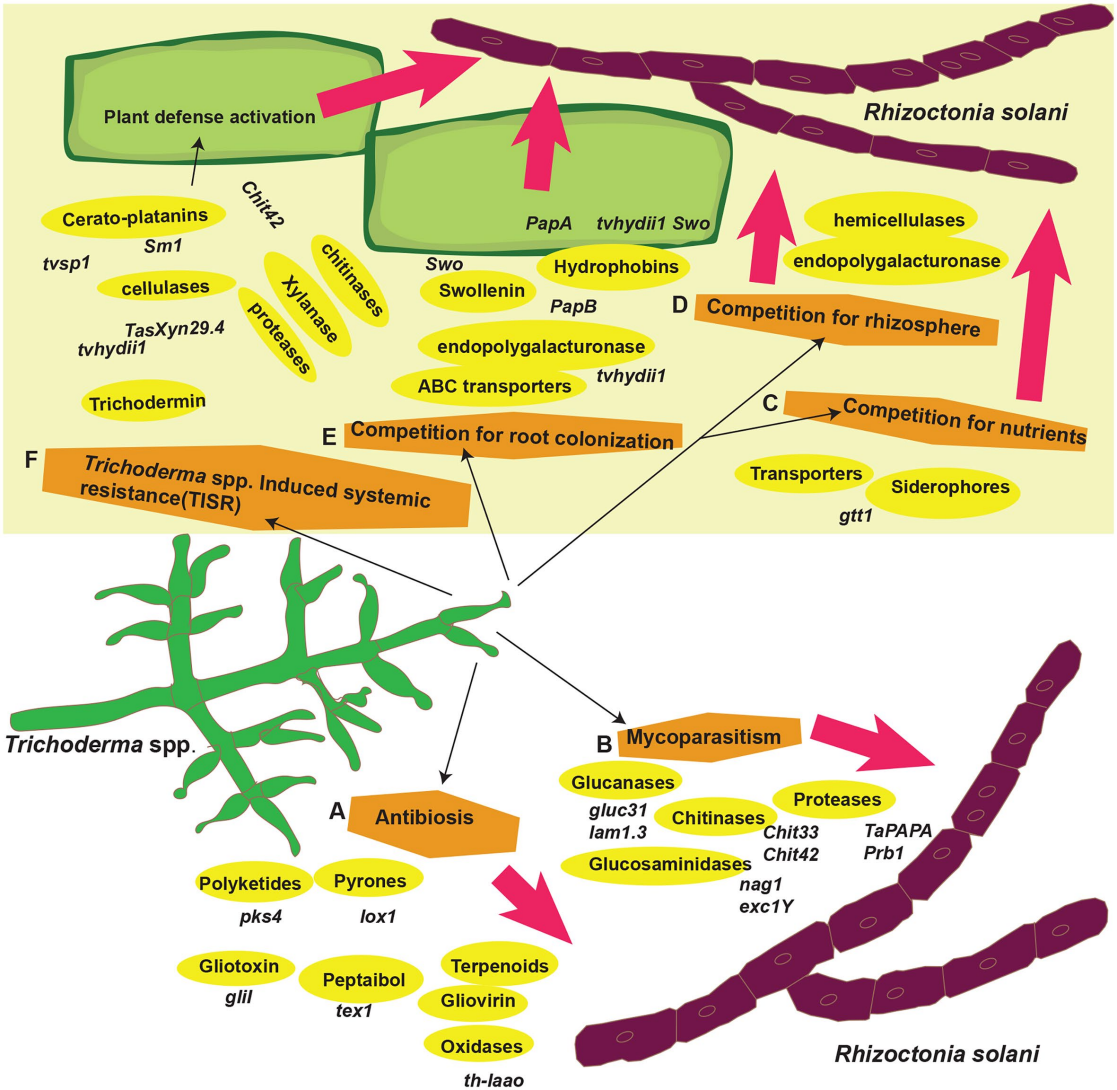
*Trichoderma* spp. biocontrol mechanisms against *R. solani*. (A) *Trichoderma* spp. genes in antibiosis, (B) mycoparasitism, (C–E) competition for root colonization, rhizosphere and nutrients, and (F) induced systemic resistance.

*Trichoderma* spp. (teleomorph: *Hypocrea*) is a saprotrophic fungus. They may survive in a variety of settings, including soil, wood, bark, other fungus, and many more, demonstrating their adaptability and opportunistic potential (Jaklitsch, 2009; Brotman et al., 2010; Druzhinina et al., 2011; Oszust et al., 2020; Vinale and Sivasithamparam, 2020; Yu et al., 2022). *Rhizoctonia solani* J.G. Kühn [teleomorph: *Thanatephorus cucumeris* (A.B. Frank) Donk], is a soil-borne pathogen with a necrotrophic lifestyle that lives in soil by developing a resistant survival structure known as *sclerotia* (Mayo et al., 2015). This fungus is a species complex that causes significant harm to numerous economically important agricultural, horticultural, pasture crops, turf grasses, and forest and fruit trees worldwide (Ajayi-Oyetunde and Bradley, 2018). It caused sheath blight in corn (Li et al., 1998) and rice (Abbas et al., 2021), seed, stem, collar, root, and hypocotyl rot and damping off in soybean, tomatoes, eggplant, pepper, lettuce, and zinnia (Ohkura et al., 2009) and stem canker and black scurf in potatoes (Das et al., 2014). In addition, the fungus caused root, stem, root, crown, and hypocotyl rot and blights of legumes and cotton (Nerey et al., 2010), grey leaf spot, and brown patch of turf grasses (Dong et al., 2008). Geographical distribution of *R. solani* has been shown in Figure 1. Chemical fungicides are widely used to control this disease, as no resistance resources have yet been discovered in available rice germplasm. Moreover, the species complex of *R. solani* is composed of various 14 anastomosis groups (AGs; AG1-13 and AG-B1), having wide genetic diversity, broad host compatibility, and ability to survive from one crop season to the next by forming dormant sclerotia made disease control even more difficult (Patil and Solanki, 2016; Abbas et al., 2021). Furthermore, agriculture confronts enormous challenges in providing enough food in a sustainable manner for an ever-increasing worldwide population while simultaneously dealing with unpredictable global environmental changes. As a result, there is an increased demand for ecologically friendly solutions that may assist plants in performing well in a range of conditions. In this regard, *Trichoderma* spp. might serve as a model fungus for sustaining agricultural output (Abdel-lateif, 2017; Singh et al., 2021). Many studies have indicated that biological management with the genus *Trichoderma* effectively controls *R. solani* (Solanki et al., 2011; Kashyap et al., 2020). For instance, in several countries, *T. harzianum* and *T. asperellum* were employed to prevent damping off, root, and crown rots (Herrera et al., 2020; Khadka and Miller, 2021). Similarly, there is a widespread use of *T. harzianum* to suppress black scurf, sheath blight, and stem canker in potatoes throughout the world (Wilson et al., 2008; de França et al., 2015; Naeimi et al., 2020). *Trichoderma* spp. can parasitize and compete with *R. solani* for nutrients, rhizosphere, and root colonization (Guzmán-Guzmán et al., 2017; Vinale and Sivasithamparam, 2020; Segreto et al., 2021; Yu et al., 2022). They can also compete for seed exudates, which stimulate the development of *R. solani* propagules in the soil (Nawrocka et al., 2018). Furthermore, *Trichoderma* spp. are prolific makers of secondary metabolites, such as peptaibols, pyrones, non-ribosomal peptides (NRPs), polyketides and terpenoids, and siderophores, when grown with *R. solani* (Manganiello et al., 2018; Halifu et al., 2020). Furthermore, they block or degrade pectinases and other enzymes required for *R. solani* development (Kullnig et al., 2000; Halifu et al., 2020). Furthermore, by colonizing the rhizospheres of plants, they drive plant development and defense responses against *R. solani* (Nawrocka et al., 2018; Zhang and Zhuang, 2020). Furthermore, *Trichoderma* spp. can activate plant se mechanisms, resulting in ISR, SAR, and, according to a new research, *Trichoderma*-induced systemic resistance (TISR; Leonetti et al., 2017). Application of *Trichoderma* spp. led to activation of defense signaling involving SA and/or JA/ET pathways against *R. solani*, hence increasing plant resistance. Recent research has produced a wealth of knowledge on the discovery and cloning of several genes involved in mycoparasitism, resistance, antibiosis, and competition induction in plants against *R. solani* (Druzhinina et al., 2011; Rahimi Tamandegani et al., 2020; Zhang and Zhuang, 2020). Numerous reviews explain the role of *Trichoderma* spp. genes against many plant pathogens (Harman et al., 2004; Zeilinger and Omann, 2007; Druzhinina et al., 2011; Nawrocka and Małolepsza, 2013; Daguerre et al., 2014; Sarma et al., 2014; Contreras-Cornejo et al., 2016; Guzmán-Guzmán et al., 2019). This article examines *Trichoderma* spp. genes that encode proteins linked with antibacterial action against *R. solani* and resistance induction in plants. The function of *Trichoderma* spp. genes in competition for root colonization, mycoparasitism, antibiosis, rhizosphere and nutrients, and stimulation of plant defensive mechanisms against *R. solani* is depicted in Figure 2. The following parts have been included in the review: (1) *Trichoderma* spp. biology, (2) genes involved in mycoparasitism, (3) genes involved in competition, (4) induced resistance in various plants against *R. solani*, and (5) resistance to *R. solani* in plants is mediated by biochemical changes associated to *Trichoderma*-induced defensive responses.

## Biology of *Trichoderma* spp.

*Trichoderma* spp. exist in two separate morphological and physiological stages. *Hypocrea* is the sexual (teleomorphic) stage, while *Trichoderma* is the asexual (anamorphic or mitosporic) stage (Rahimi Tamandegani et al., 2020; Zhang and Zhuang, 2020)*. Trichoderma* spp. that can no longer reproduce sexually are known as “agamospecies,” but sexual forms make up the majority of the genus’ genetic diversity. There is an average of 10^1^–10^3^ culturable *Trichoderma* spp. per gram of temperate and tropical soils (Harman et al., 2004; Zeilinger and Omann, 2007; Druzhinina et al., 2011; Nawrocka and Małolepsza, 2013; Daguerre et al., 2014; Sarma et al., 2014; Morán-Diez et al., 2021). These fungi also colonize plant materials, such as wood and herbaceous plants, where the sexual genus *Hypocrea* is most commonly seen. The majority of biocontrol strains lack a documented sexual stage (Druzhinina et al., 2011; Daguerre et al., 2014). Asexual fungi are clonal, frequently heterokaryotic individuals and communities that most likely evolved separately during the asexual stage (Contreras-Cornejo et al., 2016). They have a lot of genetic diversity and can make various commercial and ecologically valuable goods. They are prolific extracellular protein makers, well known for producing enzymes that digest cellulose and chitin (Harman et al., 2004; Druzhinina et al., 2011; Daguerre et al., 2014). Distinct strains, for example, produce over 100 different compounds with recognized antibiotic properties. *Trichoderma* spp. has long been known for suppressing plant disease and promoting plant growth and development. In horticulture, they are getting increasingly popular because of their “rhizosphere competence” and can colonize and develop near plant roots. Much of the known biology of these fungi and many of their uses have only lately been documented. These fungi’s biology is being considerably revised because many new species are being recognized. Most of the distinctive species in the genus *Trichoderma* were difficult to distinguish morphologically. As a result, a polyphasic approach is utilized to discover the characteristics of a novel species by combining the results of numerous techniques, such as molecular, morphological, genomic, and physiological study (Badaluddin et al., 2018). For example, one of the most common genetic methods for identifying *Trichoderma* spp. is multi-gene phylogeny. Currently, the combination of multi-gene phylogeny and morphological features is employed to identify the species level description of *Trichoderma*. Both morphological and molecular analysis employing genes including *rpb2*, *cal*, *act*, *tef1*, and ITS were used to identify *Trichoderma* spp. strains. The many functional groups within *Trichoderma* spp. that are important for secondary metabolite production were also identified utilizing a combination of novel genomic approaches and physiological activities (Zeilinger et al., 2016; Ali et al., 2021). These integrated methodologies contributed to the need to identify *Trichoderma* strains as biological control agents (Mahr, 2021). Furthermore, genomic investigations of *Trichoderma* spp. have highlighted the fungal kingdom’s genetic variety as well as distinctions in shape, physiology, and ecology (Inglis et al., 2018). Because of advancements in high-throughput sequencing technology, the number of available fungal genome data is quickly increasing. Recently, the genomes of the most common *Trichoderma* spp. were compared in an attempt to better understand *Trichoderma* biology (Chung et al., 2021).

## *Trichoderma* spp. Genes Involved in Mycoparasitism

The combination of *Trichoderma* spp. with other fungi is referred to as necrotrophic hyperparasitism or mycoparasitism. Many studies showed that *Trichoderma* spp. exhibited mycoparasitic capacity against *R. solani* (Geremia et al., 1993; De La Cruz et al., 1995; Lorito et al., 1998; Kubicek et al., 2011; Dubey et al., 2021). *Trichoderma* spp. on the other hand, feeds on fungal biomass; hence, classified as mycotrophic to encompass both saprotrophic and biotrophic feeding methods. Antibiosis, mycoparasitism, nutrient competition, rhizosphere and root colonization, and activation of plant defense mechanisms are all used by *Trichoderma* spp. to battle pathogenic fungus (Rai et al., 2019; Morán-Diez et al., 2021; Sánchez-Cruz et al., 2021; Segreto et al., 2021; Yu et al., 2022). *Trichoderma* spp. specifically detect and establish an antagonistic relationship with *R. solani*. Finally, they kill or control *R. solani* by genetic reprogramming of their gene expression. These two processes are crucial because they impact the type and degree of the *Trichoderma* spp. hostile behavior against *R. solani* (Yu et al., 2022).

### Chemotropism and Recognition of Prey

The initial phase in mycoparasitism is *Trichoderma* spp. detecting or identifying *R. solani* as prey. *Trichoderma* spp. recognize the oligopeptide and oligosaccharide compounds produced by *R. solani* in reaction to hydrolytic enzymes, such as proteases and chitinases (Druzhinina et al., 2011; Sood et al., 2020). A signaling cascade is activated when these molecules attach to receptors on *Trichoderma* spp. hyphae. This causes transcription factors to be activated, which govern production of secondary metabolite (SM) production and lysis of cell wall (Harman et al., 2004; Druzhinina et al., 2011; Table 1). Many *Trichoderma* spp. express genes encoding proteases and oligopeptide transporters before and during interaction with *R. solani*, according to a recent study. The bulk of proteases are subtilisin-like serine proteases, and genes for these enzymes may be found in abundance in expressed sequence tags (ESTs). For example, a review of the ESTs collected at the start of the *T. atroviridis*-*R. solani* interaction revealed many genes that produce subtilisin-like serine proteases. The *prb1* gene encodes these proteases, and overexpression of these proteases boosted mycoparasitic activity. The action of these proteases on *R. solani* may result in the release of oligopeptide molecules that bind to receptors on *Trichoderma* spp. Using *T. atroviride* EST libraries, a preliminary transcriptome research revealed considerable alterations in *T. atroviride* gene expression, including changes that resembled a response to nitrogen restriction, lipid metabolism adjustments, and signaling changes (Seidl et al., 2009; Kaur et al., 2021).

**Table 1:** Role of *Trichoderma* spp. genes involved in recognition of *R. solani* and signal transduction.

Protein/molecule	Receptors	Gene	*Trichoderma* spp.	References
Receptor proteins	Seven-transmembrane receptor Gpr1	*gpr1*	*T. atroviride*	Atanasova et al., 2018
	Seven-transmembrane receptor Gpr1	*gpr1*	*T. atroviride*	Omann et al., 2012
G Proteins	G Protein one	*N.A.*	*T. asperellum*	Liu et al., 2010
	G Protein ypt3	*N.A.*	*T. asperellum*	Liu et al., 2010
	G Protein rab2	*N.A.*	*T. asperellum*	Liu et al., 2010
	α-Subunit of G protein 1	*tga1*	*T. atroviride*	Rocha-Ramírez et al., 2002; Reithner et al., 2005
	G Protein-coupled receptors		*T. virens*	Halifu et al., 2020
	α-Subunit of G protein 3	*tga3*	*T. atroviride*	Zeilinger et al., 2005
Mitogen-activated protein kinases	MAPK A	*tmkA*	*T. virens*	Mukherjee et al., 2003
	MAPK 1	*tmk1*	*T. atrovide*	Reithner et al., 2007
		*tvk1*	*T. virens*	Mendoza-Mendoza et al., 2003
Others proteins	Adenylate cyclase Tac1	*tac1*	*T. virens*	Mukherjee et al., 2007
Transcription factors	pH Regulator PacC	*pacC*	*T. virens*	Trushina et al., 2013
	pH Regulator Pac1	*pac1*	*T. harzianum*	Moreno-Mateos et al., 2007
	Transcription factor ThCtf1	*ctf1*	*T. harzianum*	Rubio et al., 2009
	Velvet protein Vel1	*vel1*	*T. virens*	Mukherjee and Kenerley, 2010
	Xylanase transcriptional regulator Xyr1	*xyr1*	*T. atrovide*	Reithner et al., 2014
	Sur7 family protein	*Sfp2*	*T. atroviride*	Atanasova et al., 2018
Target of rapamycin	TOR kinase	*tsc1*	*Trichoderma atroviride*	Segreto et al., 2021

N.A., not available.

### G Protein-Coupled Receptors

**Figure 3:**
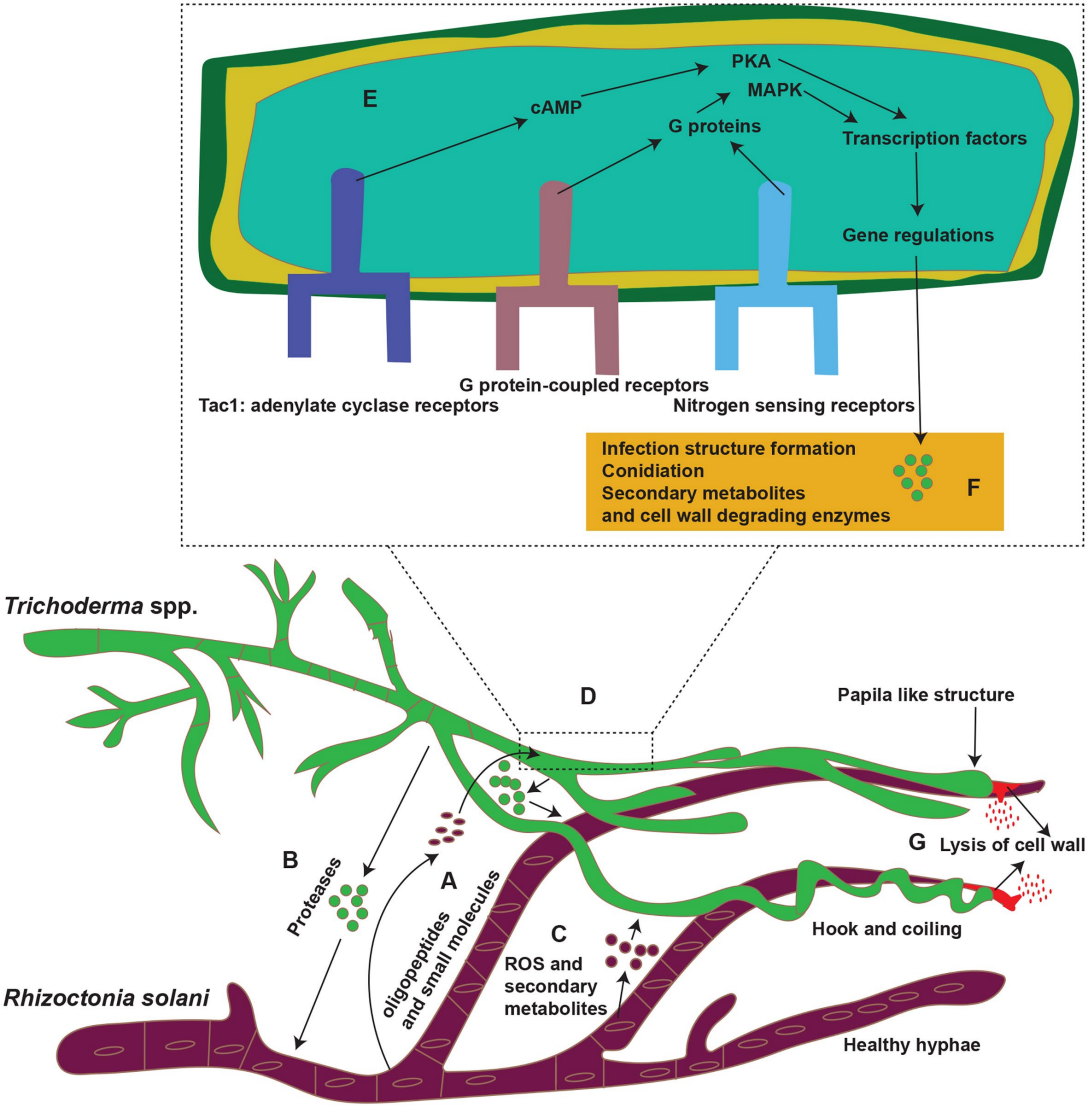
Mycoparasitism; *Trichoderma* spp. (Green color) parasitize *R. solani* (purple) in soil. (A–C) *Trichoderma* spp. recognized *R. solani* by tiny molecules (oligopeptides and small other molecules); some of these molecules are peptides released by the action of proteases of *Trichoderma* spp. prior to contact. Also *R. solani* secrete ROS and secondary metabolites in response to *Trichoderma* spp. (D) These molecules bind to G protein-coupled receptors (GPCRs; such as *Gpr1*) or nitrogen-sensing receptors (Target of rapamycin; TOR pathway), or adenylate cyclase receptors on the surface of *Trichoderma* spp. hyphae. (E) After binding to the receptors, the molecules induce a signaling cascade involving G proteins and mitogen-activated protein kinases (MAPKs) or protein kinases (PKA), which then modulate the activities of transcription factors (TFs) and gene regulations. (F,G) These substances then boost the expression of genes that code for enzymes involved in secondary metabolite production and lysis of the cell wall of *R. solani*. Reconstructed from Druzhinina et al. (2011).

G protein-coupled receptors (GPCRs) of biocontrol agents act as a sensor for the oligopeptides secreted by plant pathogens (Daguerre et al., 2014). They are the most frequent cell surface receptors for detecting environmental signals at the plasma membrane. These receptors sense ligands, such as nutrients, oligopeptides, sex pheromones, oxylipins. They frequently use heterotrimeric G proteins to connect with downstream signaling pathways including the mitogen-activated protein kinase (MAPK) and cAMP-protein kinase A (PKA) cascades (Table 1; Figure 3). In response to *R. solani*, several GPCR-encoding genes were expressed in *Trichoderma* spp. showing that GPCRs play a role in detecting and triggering the mycoparasitic response. The three primary components of heterotrimeric G protein signaling are a GPCR, a heterotrimeric G protein (made up of G and G subunits), and an effector. G protein activation (exchange of GDP for GTP on the G subunit) and dissociation of the GTP-bound subunit from the associated dimer occur as a result of the ligand contact, allowing both units to govern downstream effectors. In fungus, the cAMP-PKA pathway and MAPK cascades are typical effectors of heterotrimeric G protein. Both routes are substantially conserved and linked. Transmembrane G protein-coupled receptor signals are channeled by heterotrimeric G proteins to various intracellular destinations *via* activating effectors, such as adenylate cyclase or the MAPK cascades (Kaziro et al., 1991; Daguerre et al., 2014; Segreto et al., 2021). *Trichoderma* spp. require G proteins, GPCRs, and adenylate cyclase receptors to synthesize external cell wall lytic enzymes, release antifungal chemicals, and produce infection structures (Figure 3). *Trichoderma* hyphae were prevented from connecting to *R. solani* cell surfaces by inhibiting the gene encoding the *T. atroviride* seven-transmembrane receptor *Gpr1*, as well as upregulation of two chitinase genes (*nag1* and *ech42*) and the protease gene *prb1*. Due to the importance of these genes in mycoparasitism, their downregulation in *T. atroviride* results in pathogen survival (Omann et al., 2012). Two G protein subunits identified in *T. atroviride* are *Tga1* and *Tga3*. During direct fight with *R. solani*, the *tga1* mutant completely lost its mycoparasitic activity (Reithner et al., 2011). Infection structure development remained steady, while production of 6-pentyl-pyrone and sesquiterpene-derived antifungal metabolites reduced (Rocha-Ramírez et al., 2002; Reithner et al., 2011). Similarly, when addressed directly, the *tga3* mutant was unable to build infection structures or mycoparasitize *R. solani* (Zeilinger et al., 1999). In fungus, the MAPK pathways are well-known signal transduction systems (Schmoll et al., 2016). *Trichoderma’s* genome contains genes for three pathogenicity MAPKs: (1) *TmKA* (also known as *Tvk1* and *Tmk1*), (2) cell integrity kinase (*TmkB*), and (3) osmoregulatory MAPK (*Hog1*; Schmoll et al., 2016). *TmkA* gene mutation in *T. virens* strain “P” had no effect on biocontrol efficacy against *R. solani* (Zeilinger et al., 1999; Viterbo et al., 2004; Mukherjee et al., 2011, 2012). However, deletion of the same gene *TmkA* in the *T. virens* strain “Q” drastically lowered the *Trichoderma’s* biocontrol efficacy against *R. solani* (Mendoza-Mendoza et al., 2003). *Trichoderma virens tvk1* mutants secreted more lytic enzymes and were far more efficient in disease control than the wild-type strain (Mukherjee et al., 2003). *Trichoderma atroviride tmk1* mutants displayed decreased mycoparasitism activity against *R. solani* in direct mycoparasite–host interactions, as well as against *R. solani* specific control of *ech42* gene transcription (Reithner et al., 2011). Furthermore, deletion of a *Tmk1* homologue in *T. atroviridis* resulted in decreased mycoparasitic activity against *R. solani*, as well as increased synthesis of chitinase and other antifungal chemicals (Reithner et al., 2011).

### Cyclic Adenosine Monophosphate Receptors

In addition to, GPCRs, another importance receptors for signaling transduction are Cyclic adenosine monophosphate (cAMP) receptors. They are involved in the growth, condition, development, and biocontrol efficiency in *Trichoderma* spp. (Brunner et al., 2008; Table 1; Figure 3). Adenylate cyclase converts ATP to cAMP and is present on the inner side of the plasma membrane and at several sites throughout the fungal cell. Adenylate cyclase is triggered by a multitude of signaling molecules that activate G (Gs) protein-coupled receptors that stimulate adenylate cyclase. cAMP activates a cAMP-dependent protein kinase (PKA) that phosphorylates proteins like transcription factors to regulate gene expression (Dickman and Yarden, 1999). Tac1, an adenylate cyclase gene in *T. virens*, was deleted, which not only removed biocontrol efficacy against *R. solani* but also lowered secondary metabolite synthesis (Mukherjee et al., 2007).

### Target of Rapamycin Proteins

In addition to cAMP and MAPK, the target of rapamycin (TOR) pathway is a critical regulator of *Trichoderma* spp. cell proliferation in response to nutrient availability (Table 1; Figure 3). In response to a lack of carbon and nitrogen, this pathway is triggered, resulting in anabolic activities and development (Schmoll et al., 2016). The TOR kinase is inhibited by rapamycin, and nutritional deficiency enhances the expression of genes involved in alternate nitrogen absorption. A growing body of evidence suggests that TOR has a role in nitrogen signaling and pathogenicity-related activities in fungal plant diseases. The *Trichoderma* genomes also encode single TOR kinase-like the fungal plant pathogens. In a recent study, the activity of TOR1, that is, *T. atroviride*’s solitary and crucial TOR kinase, was suppressed by chemical TOR inhibitors or genetic alteration. TSC2 and TSC1, which are negative regulators of TOR complex 1 (TORC1) in human cells, resulting in altered nitrogen source-dependent growth of *T. atroviride*, decreased generation of numerous secondary metabolites, and decreased mycoparasitic overgrowth on *R. solani* (Segreto et al., 2021). Transcription factors (TFs), which regulate gene transcription at the cellular level during antagonism, are currently understudied (Table 1). Specific motifs in the promoters of *Trichoderma* spp. biocontrol genes may bind transcription factors involved in nitrogen repression, stress responses, and the regulation of plant cell wall-degrading enzymes. There is no indication, however, that they have a role in antifungal activity. For example, *T. atroviride’s* xylanase transcriptional regular *Xyr1* has a role in mycoparasitism and is essential to trigger the plant defense response (Reithner et al., 2014). When *xyr1* was eliminated, *R. solani* competed more effectively. Plant defense responses were similarly delayed during the *T. atroviride*/*Arabidopsis thaliana* interaction.

### Attachment and Coiling

*Trichoderma* spp. coil and generate helix-shaped hyphae around *R. solani* shortly after recognition, and this phenomenon is dependent on lectin recognition from *R. solani’s* cell wall. Conversely, plant lectins also cause coiling, demonstrating that lectins do not determine specificity in *Trichoderma* spp. Additionally, coiling is not always associated with mycoparasitism, as some *Trichoderma* spp. do exhibit this characteristic. Besides, hyphae of *Trichoderma* spp. become spiral or helical in shape and are considered diagnostic features in some *Trichoderma* spp. *Trichoderma* spp. often precede mycoparasitic attack by growing alongside the host hyphae and forming papilla-like structures. At the places where papilla-like structures form, the cell wall is degraded, and the lumen is penetrated. These papilla-like structures are identical to the appressorium of plant pathogenic fungi and those generated. Recent study suggests the presence of essential components of the cAMP and MAP kinase signaling pathways, such as G protein subunits (G), which govern extracellular enzyme synthesis, antibiotic production, and coil formation surrounding *R. solani* and *T. atroviride* expressed the G-gene (tga1) under the control of its promoter or the promoter of the proteinase gene (prb1; Reithner et al., 2005). All mutants showed an increase in coiling. Furthermore, *T. viride* overexpressing tga1 exhibited a greater capacity to outgrow *R. solani*. Induction of genes encoding ABC efflux transporters, pleiotropic and multidrug resistance transporters, and detoxification mechanisms (such as those encoding ABC efflux transporters and pleiotropic and multidrug resistance transporters) and detoxification mechanisms (such as those encoding ABC efflux transporters and pleiotropic and multidrug resistance transporters) in the presence of *R. solani* is a distinctive feature of *Trichoderma* spp. When *R. solani* develops sclerotia, it signals with radical oxygen species and excretes antifungal chemicals into the environment. Both radical oxygen species and antifungal drugs have been demonstrated to promote *Trichoderma* spp. stress’s response. The deletion of one of the genes in *T. atroviridis* that produces an ABC transporter (Abc2) resulted in poor biocontrol of *R. solani*, demonstrating that detoxification plays a role in mycoparasitism.

### Death of the Fungus

Secondary antifungal metabolites, such as NRPs (peptaibols, gliotoxin, gliovirin, etc.), polyketides, isoprenoid-derived metabolites, pyrones, and cell wall-hydrolytic enzymes or degrading enzymes (CWDEs) eventually kill the prey (Table 2; Figure 3). *Trichoderma* spp. genomes contain many genes for the synthesis of antifungal metabolites and CWDEs. *Trichoderma virens*, for example, possess the most non-ribosomal peptide synthesis of any plant pathogenic fungus. The cell wall of any fungus is composed of 30% dry weight chitin, -1,3-glucans, -1,3-glucans, and -1,4-glucans. Cellulases, polygalacturonases (PG), chitinases, glucanases, and proteinase are only a few of the CWDEs found in *Trichoderma* spp.

**Table 2:** Role of *Trichoderma* spp. genes in the mycoparasitism of *R. solani*.

Protein/molecule	MW kDa	Gene	*Trichoderma* spp.	References
Endochitinases (GH 18)	Chitinase 1	*N.A.*	*T. asperellum*	Liu et al., 2010
	33-KDa Endochitinases	*chit33*	*T. harzianum*	De las Mercedes Dana et al., 2001
		*ech33*		Sharma and Bhat, 2011
		*Tv-cht1*	*T. virens*	Kim et al., 2002
		*Tv-cht2*	*T. virens*	Kim et al., 2002
	36-KDa Endochitinases	*chit36Y*,	*T. asperellum*	Viterbo et al., 2001
	42-KDa Endochitinases	*chit42*	*T. atroviride*	Kullnig et al., 2000
		*echi42*	*T. asperellum*	Carsolio et al., 1999
		*chit42*	*T. harzianum*	Kullnig et al., 2000
		*ech42*	*T. harzianum*	Liu et al., 2010
		*chit42*	*T. harzianum*	Zeilinger et al., 1999
		*Tv-ech1*	*T. virens*	Baek et al., 1999
		*Tv-ech2*	*T. virens*	Kim et al., 2002
	46-KDa Endochitinase	*chit46*	*T. asperellum*	Lima et al., 1997
	Endochitinases (GH 18)	*crchi1*	*T. harzianum*	Limón et al., 1995
Glucosaminidases (GH 20)	N-Acetyl-β-glucosaminidases	*exc1Y*	*T. asperellum*	Viterbo et al., 2002
		*nag1*	*T. atroviride*	Brunner et al., 2003
		*eng18B*	*T. atroviride*	Dubey et al., 2012
		*Tvnag1*	*T. virens*	Kim et al., 2002
		*Tvnag2*	*T. virens*	Kim et al., 2002
Glucanases	β-1,3-Glucanases	*tag83*	*T. asperellum*	Marcello et al., 2010
		*lam1.3*	*T. harzianum*	Marcello et al., 2010
		*gluc31*		Suriani Ribeiro et al., 2019
	29-KDa b-1,3-Glucanase	*N.A.*	*T. harzianum*	Noronha et al., 2000
	36-KDa b-1,3-Glucanase	*N.A.*	*T. harzianum*	Noronha et al., 2000
	78-KDa b-1,3-Glucanase	*bgn13.1*	*T. harzianum*	De La Cruz et al., 1995
	β-1,6-Glucanase	*bgn16.2*	*T. harzianum*	De La Cruz et al., 1995
		*Tvbgn3*	*T. virens*	Djonović et al., 2007
	β-1,3-Glucanase	*N.A.*	*T. koningii*	Kim et al., 2002
		*Tvbgn1*	*T. virens*	Kim et al., 2002
		*Tvbgn2*	*T. virens*	Kim et al., 2002
	Endo-1,3(4)-β-glucanase	*N.A.*	*T. asperellum*	Liu et al., 2010
Proteases	Aspartic proteases	*TaAsp*	*T. asperellum*	Yang et al., 2013
		*TaPAPA*	*T. asperellum*	Viterbo et al., 2004
		*Sa76*	*T. harzianum*	Liu and Yang, 2007
		*P6281*	*T. harzianum*	Suárez et al., 2005
		*PAPA*	*T. harzianum*	Delgado-Jarana et al., 2002
	Serine proteases	*Spm1*	*T. asperellum*	Liu et al., 2010
		*tvsp1*	*T. virens*	Pozo et al., 2004
		*prb1*	*T. harzianum*	Geremia et al., 1993
		*pra1*	*T. harzianum*	Reithner et al., 2011
		*papA*	*T. atroviride*	Reithner et al., 2011
		*papB*	*T. asperellum*	Viterbo et al., 2004
Miscellaneous	CoA reductase	*hmgR*	*Trichoderma* spp.	Gajera et al., 2016
	Mitogen-activated protein kinase	*task1*	*T. asperellum*	Yang, 2017
	Pore-forming proteins	*agl1*	*T. atroviride*	Dubey et al., 2021

N.A., not available.

#### Chitinases

Several chitinases enzymes are found in *Trichoderma* spp., and the list of these enzymes is updated continuously as new enzymes and their associated genes are discovered. *Trichoderma* spp. produce both endo and exochitinases that belong to the glycosyl hydrolase (GH) family. GH is divided into three groups based on amino acid sequence similarity: GH 18, GH 19, and GH 20 (Kim et al., 2002). Endochitinases break chitin into chitotetraose, chitotriose, and diacetylchitobiose at internal locations. Chitobiosidases and N-acetyl-glucosaminidases were classified further into exochitinases. Chitobiosidases are enzymes that catalyze the stepwise release of diacetylchitobiose. Diacetylchitobiose is broken into N-acetylglucosamine monomers by N-acetylglucosaminidases (Baek et al., 1999). These chitinases degrade chitin polymers by breaking β-1,4 glycosidic linkages in the hyphae of *R. solani*. Many chitinase-encoding genes have recently been found and reported, and their antagonistic action against *R. solani* has been tested (Table 2). *Trichoderma harzianum* and *T. atroviride* have the most widely explored chitinolytic system among the *Trichoderma* spp. The biocontrol activity of *T. virens* transformants overexpressing *Cht42* against *R. solani* in cotton seedling tests was greatly increased as compared to the wild type, as demonstrated by the results of previous investigations (Baek et al., 1999). When the same gene was expressed in other *Trichoderma* spp. it resulted in higher biocontrol activity against *R. solani* than when the wild type was used (Howell, 2003). However, in greenhouse biocontrol testing, the activity of chit42 mutants was identical to that of the wild type (Harman et al., 2004). Limón et al. (2004) identified and reported transformants of the biocontrol agent *T. harzianum* strain CECT 2413 that overexpressed a 33 kDa chitinase (chit33). Under the guidance of the *T. reesei pki* constitutive promoter, strain CECT 2413 was co-transformed with the amdS gene and its chit33 gene. The transformants were more effective in inhibiting *R. solani* growth than the wild type (Limón et al., 2004).

#### Glucanases

In synergistic cooperation with chitinases and secondary metabolites, glucanases have been demonstrated to reduce spore germination or pathogen growth. Glucans are glucose polysaccharides that act as crosslinks between chitin or chitosan polymers. There are two types of glucans, which are distinguished by the chemical link that exists between the glucose subunits. The stiffness of the cell wall is provided by β-glucans, which are made up of -(1,3)- or -(1,6)-linkages. In contrast, α-glucans are made up of -(1,3)- and/or -(1,4)-linkages and serve as a matrix component. Many glucanases with antagonistic activity against *R. solani* have been isolated from *Trichoderma* spp. as shown in Table 2. These enzymes degrade glucan polymers in *R. solani* hyphae by cleaving β-1,3 glycosidic linkage. When the gene *bgn13.1* was overexpressed in *T. harzianum*, it resulted in the greatest suppression of *R. solani* infection. A higher level of antagonistic activity was seen in the case of the oomycete, *P. citrophthora*, which has cellulose and glucans as its primary cell wall components, compared to the *R. solani*, which has chitin and glucan as its primary cell wall components. Many 1,6-glucanases have also been isolated from *Trichoderma* spp. and have demonstrated antagonistic activity, either alone or in conjunction with chitinases (da Silva Aires et al., 2012). It has recently been shown that *T. harzianum* strain ALL42 contains a gene that encodes an endo-1,3-glucanase that is related to the GH16 family, and that this gene is involved in the metabolism of glucans. The lack of the *gluc31* gene had no effect on the *in vivo* mycoparasitism capacity of mutant *T. harzianum* ALL42 against *R. solani*; however, the removal of the *gluc31* gene appeared to have an impact on the structure of the cell wall of *T. harzianum* ALL42 (Suriani Ribeiro et al., 2019).

#### Proteases

There are several varieties of fungal proteases (also known as fungal peptidases or proteolytic enzymes) that help in the lysis of cell walls (Mata-Essayag et al., 2001; Haggag et al., 2006). They accelerate the peptide bond breakage in other proteins. Fungal proteases are peptide hydrolases or peptidases that belong to a large number of enzymes that may be divided into endopeptidases and exopeptidases. Several investigations have shown that *Trichoderma* spp. exopeptidases contribute in the biocontrol of *R. solani* (Table 2). In addition to breaking down the host cell wall, fungal proteases may function as proteolytic inactivators of pathogen enzymes involved in plant infection (Suárez et al., 2005). Table 2 shows the mycoparasitic protease genes of *Trichoderma* spp. that have been cloned so far. They also encode aspartic and serine proteases that function in the same way as subtilisin, chymotrypsin/elastase, and trypsin (Pozo et al., 2004; Yang, 2017). Prb1 from *T. harzianum* IMI 206040 has been shown to play an essential role in biological control, and prb1 transformants increased the biocontrol effectiveness of *Trichoderma* strains against *R. solani* by up to fivefold (Flores et al., 1997; Herrera-Estrella, 1997; Cortes et al., 1998; Goldman and Goldman, 1998). *Trichoderma harzianum’s* protease pra1 has a preference for fungal cell walls. *T. virens* extracellular serine protease gene (tvsp1) was cloned, and its overexpression dramatically enhanced cotton seedling protection against *R. solani* (Pozo et al., 2004). Another study found that cold-tolerant *T. harzianum* strains produced chitinases, glucosidases, trypsin-like, and chymotrypsin-like proteases that were active at low temperatures (Antal et al., 2000; Szekeres et al., 2004). Furthermore, it was shown that mutants obtained by UV irradiation produced substantially more proteases. Some of these mutants have been found to be effective *R. solani* antagonists. According to a recent RNA sequencing study, 20 genes associated with mycoparasitism, including extracellular proteases, oligopeptide transporters, GPCRs, chitinases, glucanases, and proteases, were found to be upregulated during the antagonistic process between *T. virens* ZT05 and *R. solani* (Halifu et al., 2020).

## *Trichoderma* spp. Genes Involved in the Antibiosis

Antibiosis is the antagonism of *R. solani* caused by the toxicity of secondary metabolites generated by *Trichoderma* spp. In *Trichoderma* spp. several genes involved in secondary metabolite synthesis have been discovered (Cardoza et al., 2007; Ruocco et al., 2009; Vinale and Sivasithamparam, 2020). As indicated in Table 3, these genes encode secondary metabolites, such as pyrones, polyketides, peptaibols, gliotoxin, gliovirin, terpenoids, and other chemicals. Depending on the chemical and the target location, varying amounts of these compounds are poisonous to *R. solani* (Malmierca et al., 2013; Rahimi Tamandegani et al., 2020).

**Table 3:** Role of *Trichoderma* spp. genes involved in the antagonism and synthesis of secondary metabolites deleterious to *R. solani.*

Pathways	Protein/molecule	Gene	*Trichoderma* spp.	References
Pyrone biosynthesis pathway	Lipoxygenase	*lox1*	*T. atroviride*	Kubicek et al., 2011; Speckbacher et al., 2020
	6-Pentyl-α-pyrone (6-PP)	*N.A.*	*Trichoderma* spp.	Kotasthane et al., 2015
Polyketide biosynthesis pathway	Polyketide synthases (PKS)	*pks4*	*T. reesei*	Atanasova et al., 2013b
		*pks4*	*T. virens*	Atanasova et al., 2013b
		*pks4*	*T. atroviride*	Atanasova et al., 2013b
Peptaibol biosynthesis pathway	Non-ribosomal peptide synthetases (NRPS)	*tex1*	*T. asperellum*	Rahimi Tamandegani et al., 2020
Gliotoxin and gliovirin biosynthesis pathway	Aminotransferase	*gliI*	*T. virens*	Atanasova et al., 2013a
	GliC Cytochrome P450	*gliC*	*T. virens*	Atanasova et al., 2013a
	GliC Cytochrome P450	*gliF*	*T. virens*	Atanasova et al., 2013a
	ɣ-Glutamyl cyclotransferase-like protein	*gliK*	*T. virens*	Atanasova et al., 2013a
	Glutathione S-transferase	*gliG*	*T. virens*	Atanasova et al., 2013a
	Methyltransferase	*gliN*	*T. virens*	Atanasova et al., 2013a
	NRPS modules	*gliP*	*T. virens*	Atanasova et al., 2013a
	O-Methyltransferase	*gliM*	*T. virens*	Atanasova et al., 2013a
Terpenoid/steroid synthesis pathway	Cytochrome P450 monooxygenases	*tri4*	*T. arundinaceum*	Malmierca et al., 2012
	Hydroxy-methylglutaryl-CoA reductase	*hmgR*	*T. harzianum*	Cardoza et al., 2007
	Major facilitator superfamily transporter	*Thmfs1*	*T. harzianum*	Liu et al., 2012
	Trichodiene synthase	*tri5*	*T. arundinaceum*	Malmierca et al., 2013
Oxidases	L-Amino acid oxidase	*Th-LAAO*	*T. harzianum*	Yang et al., 2011
Other’s proteins	4-Phosphopantetheinyl transferase	*ppt1*	*T. virens*	Cheng et al., 2011
Transporters	ABC transporters	*Taabc2*	*T. atroviride*	Ruocco et al., 2009
Miscellaneous	CoA reductase	*hmgR*	*T. koningii*	Gajera et al., 2016
	Mitogen-activated protein kinase	*task1*	*T. asperellum*	Yang, 2017
	Harzianic acid (HA)		*T. harzianum*	Manganiello et al., 2018
	Helicase-related proteins	*ipa-1*	*Trichoderma virens*	Estrada-Rivera et al., 2020
	p450 Monooxygenases	*TvCyt2*	*Trichoderma virens*	Ramírez-Valdespino et al., 2018

### Pyrones

Pyrones are a good example of secondary metabolites produced by *Trichoderma* spp. that have strong biocontrol activity against *R. solani*. They have numerous antagonistic activities against *R. solani* were isolated from several *Trichoderma* spp. For example, a characteristic aromatic odor resembling coconut was observed in a *T. harzianum* and *T. viride* due to 6-pentyl-α-pyrone (6-PP). Pyrones with significant antifungal action against *R. solani* were observed as one of the paramount secondary metabolites produced by *Trichoderma* spp. (Kotasthane et al., 2015).

### Polyketides and Non-ribosomal Peptides

#### Polyketides

Polyketides (PKs) are a large collection of carbon-skeletoned compounds that include polyphenols, macrolides, polyenes, enediynes, and polyethers. Their synthesis is based on the regulated assembly of acetate and propionate, notwithstanding their structural and functional diversity. *T. reesei*, *T. atroviride*, and *T. virens* have all been shown to have PKs genes with antimicrobial action against *R. solani*. Two *T. atroviride* PK_S_ genes were expressed during the encounter with *R. solani*, indicating a possible role in mycoparasitism (Mukherjee et al., 2012). Furthermore, deletion of the *PKs4* gene in *T. reesei* altered the regulation of other PKs-encoding genes and lowered antagonistic activity against *R. solani* (Atanasova et al., 2013a). According to recent comparative genomics research, *T. virens* and *T. atroviride* have a considerable number of non-ribosomal peptide (NRP) and polyketides synthases genes, with *T. virens* having more NRPs than any other filamentous fungus investigated thus far (Niu et al., 2020).

#### Non-ribosomal Peptides

Non-ribosomal peptides are produced by *Trichoderma* spp. without the involvement of ribosomes or messenger RNAs by multidomain mega-enzymes called non-ribosomal peptide synthetases (NRPSs). NRPs perform a variety of biological functions, including iron uptake, antibacterial, and antifungal activity. Peptaibols synthesis against *R. solani* is attributed to NRPSs, which construct a variety of compounds from a variety of precursors, including non-proteinogenic amino acids and hydroxy or carboxyl acids (Mukherjee et al., 2011, 2012). However, NRPSs genes from additional biological control agents have yet to be characterized.

##### Peptaibols

Peptaibols are antibacterial, antifungal, and antiviral short-chain linear polypeptides (Mukherjee et al., 2011). *Trichoderma* spp. secrete peptaibols in a combination of isoforms with over 300 sequences discovered so far (Szekeres et al., 2004). Peptaibols biological activity stems from their membrane-modifying abilities, ability to make holes in lipid membranes, and proclivity to establish systemic resistance in plants against plant diseases, such as *R. solani* (Vey et al., 2001). So far, one NRPS generated by the tex1 gene has been identified as playing a role in peptaibols synthesis in *Trichoderma* spp. in response to *R. solani* (Table 3; Rahimi Tamandegani et al., 2020).

### Gliotoxins

Gliotoxins are mycotoxins that contain sulfur have antimicrobial, antiviral, and immunomodulatory activities (Vey et al., 2001). The cell wall-degrading enzymes of *Trichoderma* spp. augment their antifungal activity synergistically (Lorito et al., 1998). Several genes of *T. virens* that encode gliotoxins, such as *gliI*, *gliC*, *gliF*, and *gliG*, appear to be important in antifungal action against *R. solani* (Atanasova et al., 2013a,b). However, these genes transcription is regulated by a number of factors, including pH, temperature, culture medium composition, and aeration (McDonagh et al., 2008; Table 3).

### Terpenes

Terpenes are natural compounds having the formula (C_5_H_8_). There are various classes of terpenes and classification is based on the number of carbon (C) atoms as; C15; sesquiterpenes, C5; hemiterpenes, C20; diterpenes, C10; monoterpenes, C30; triterpenes, C40; tetraterpenes, C25; sesquiterpenes, or polyterpenes (Daguerre et al., 2014). In *Trichoderma* spp. a gene *hmgR* that codes for hydroxy-methylglutaryl-coenzyme has been identified. An enzyme reductase (HMGR) converts hydroxy-methylglutaryl-coenzyme into mevalonate. Mevalonate is than required for the formation of terpene compounds, such as terpene cyclases, trichodermin, triterpene viridin, and trichothecenes (Figures 2, 3). Some of these compounds, such as trichothecenes and trichodermin produced by *Trichoderma* spp., have antifungal activity against *R. solani*. Other terpenes compounds, such as triterpene ergosterol, are required for cell membrane fluidity. *Trichoderma harzianum* antifungal activity was diminished when *hmgR* was largely silenced, demonstrating that terpenoid chemicals are important in antagonism (Cardoza et al., 2007; Table 3). *Harzianum A* is an example of trichothecene, which inhibits fungal plant infections, such as *R. solani*, and induces genes involved in plant defense. *Harzianum A* synthesis is regulated by the tri gene cluster, which was recently identified in *T. arundinaceum* (Cardoza et al., 2007; Malmierca et al., 2013). When the genes tri5 and tri4 were disrupted, the generation of *Harzianum A* ceased, and the biocontrol activity of the transformants against *R. solani* was diminished.

### Oxidases

Oxidases are crucial to *Trichoderma* spp.’ antagonistic activities against *R. solani*. During oxidation of glucose by oxidases, hydrogen peroxide (H_2_O_2_) is produced. Hydrogen peroxide inhibits sclerotia and subsequent hyphal growth of fungi when glucose is present. *T. harzianum* ETS 323 extracellular proteins recently yielded a new L-amino oxidase (Th-LAAO; Yang et al., 2011). In the presence of Th-LAAO, hyphal lysis of *R. solani* was seen *in vitro*. The efficiency of fungal antagonism against soil-borne pathogens is based not only on the production and release of antimicrobial components but also on antagonistic fungi’s capacity to protect themselves against toxins. To defend themselves from toxins generated by infections or themselves, biocontrol agents have many genes that encode ABC transporters and detoxifying enzymes. *T. atroviride* Taabc2 deletion mutants, for example, were less resistant to fungal inhibitory compounds, including their own, and performed less well in defending tomato plants against *R. solani* assaults (Ruocco et al., 2009).

## *Trichoderma* spp. Genes Involved in Competition

### Competition for Nutrients

Microorganisms require nutrients to survive, so competition for nutrient constraints and the colonization of plant tissues results in pathogens control (Sarma et al., 2014). When resources are few, microorganisms with the same ecological niche and physiological requirements struggle for nourishment (de Boer et al., 2003). *Trichoderma* spp., compete with *R. solani* for nutrients, mainly carbon (Sivan and Harman, 1991; Sarrocco et al., 2009). Comparatively to other fungi, they are better at mobilizing and absorbing soil nutrients (Sood et al., 2020). Moreover, in comparison to other fungi, they have a remarkable ability for ATP through the sugars metabolism including cellulose, hemicelluloses, glucans, and chitin (Oszust et al., 2020). Biomass components, mainly cellulose and hemicelluloses, are thought to be significant determinants of biocontrol fungi’s antagonistic activity. They are intended to be part of the saprophytic lifestyle and plant pathogen competition. *Trichoderma* spp., undoubtedly the most investigated fungal biocontrol agent, contain bulk of the genes encoding biomass-degrading hydrolytic enzymes found so far. Proteases, cellulases, hemicelluloses and amylases, are biological substrates degrading hydrolytic enzymes. As a result, *Trichoderma* spp. aid in carbon recycling. *Trichoderma* spp. and *R. solani*’s competitive capacity for cellulose utilization on wheat straw was assessed in a prior research (Sarrocco et al., 2009). Because wheat straw is the primarily source of cellulose and hemicelluloses, cellulolytic activity levels measured as mechanism in the straw possession competition. Fungus competition may also be influenced by the prompt uptake of nitrogen and carbon molecules that are either naturally present or released in the soil. Overexpression has been linked to the absorption of nutrients produced through the destruction of pathogenic fungi’s fungal cell walls, as well as direct nutrients competition in the soil, according to many transcriptome investigations. So far, only Gtt1, a high-affinity glucose transporter discovered in *T. harzianum*, has been examined, and its mRNA level rose in response to *R. solani* (Delgado-Jarana et al., 2003; Table 4). Competition for micronutrients can also arise in the soil. The most well-known example is competition for iron, which is essential for fungal pathogen development and pathogenicity. *Trichoderma* spp. secrete a number of siderophores that chelate iron and alter its availability to other bacteria. *T. harzianum* produced the most siderophores and had potent antifungal properties. A paucity of iron in the environment causes siderophore development and iron competition. *Trichoderma* spp. biocontrol ability against *R. solani* is influenced by iron competition (Table 4). As compared to *R. solani* and *Trichoderma* spp. can more effectively access the limited amounts of iron available. A peptide synthetase gene, *Psy1*, has been discovered. *Psy1* disruptants generated normal levels of gliotoxin but struggled to grow in low-iron environments, indicating that *Psy1* is involved in siderophore synthesis (Wilhite et al., 2001). Harzianic acid is a siderophore released by *T. harzianum* that promotes plant development while also acting as an antifungal against *R. solani* (Vinale et al., 2013).

**Table 4:** Role of *Trichoderma* spp. genes involved in competition for nutrients and root colonization against *R. solani.*

Competition	Protein/molecule	*Gene*	*Trichoderma* spp.	References
Root colonization	Class II hydrophobin family members,	*tvhydii1*	*T. virens*	Guzmán-Guzmán et al., 2017
	Endopolygalacturonase Thpg1	*Thpg1*	*T. harzianum*	Morán-Diez et al., 2009
Nutrients	High-affinity glucose transporter Gtt1	*Gtt1*	*T. harzianum*	Delgado-Jarana et al., 2003
Siderophores	Harzianic acid	*N.A.*	*T. harzianum*	Vinale et al., 2013
	Peptide synthetase	*N.A.*	*Trichoderma* spp.	Wilhite et al., 2001

N.A., not available.

### Competition for Rhizosphere

Ahmad and Baker (1987) coined the term rhizosphere competence for *Trichoderma* spp. which they described as the capacity of these fungi to grow and operate in the growing rhizosphere. The rhizosphere is a common ecological habitat for *Trichoderma* spp. and it provides saprotrophy and biotrophy possibilities on plant root exudates. Mucigel is a slimy gel-like capsule that covers plant root terminals (Oszust et al., 2020; Vinale and Sivasithamparam, 2020; Yu et al., 2022). The outermost cells of the root cap expel highly hydrated polysaccharides, such as pectins and hemicelluloses (arabinoxylans and rhamnogalacturonans). *Trichoderma* spp. produce hemicellulases (hemicellulolytic) and cellulases (cellulolytic) to utilize polysaccharides more effectively than *R. solani* secreted by plant root tips (Guzmán-Guzmán et al., 2017). For example, an endopolygalacturonase expressing gene, is required for the effective establishment of *T. harzianum* in the tomato rhizosphere, and this gene is also useful in root colonization and the induction of plant defenses. Plants also excrete saccharides, such as monosaccharides, disaccharides, and sucrose, which offer essential carbon substrate for *Trichoderma* spp. rhizosphere establishment (Oszust et al., 2020). *Trichoderma* spp. have also genes that encode intracellular invertases; for example, sucrose permease takes up sucrose before being hydrolyzed. *Trichoderma* spp. also have particular sucrose transporter and have biochemical properties similar to plant sucrose transporters (Sood et al., 2020). Sucrose is actively transmitted from plant to fungus, according to these shards of evidence. Furthermore, several *Trichoderma* spp. express a large number of important solute transporters, the functions of which to acquire additional root exudates are unclear (Zhang and Zhuang, 2020). In conclusion, the presence of pathogens and root-derived nutrients may have been significant attractants for *Trichoderma* spp. to establish themselves in the rhizosphere and create relationships with plant roots.

### Competition for Colonization of Intercellular Root Spaces

The ability to recognize and cling to roots, penetrate the plant, and endure toxic compounds released by the plant in reaction to invasion is necessary for root colonization. *Trichoderma* spp. colonize the intercellular spaces of the first or second layer of root cells (Brotman et al., 2008). The hyphal and conidial cell walls contain several proteins that aid *Trichoderma* spp. in attaching to the roots *via* appressorium-like structures (Steindorff et al., 2012; Table 4). Enzymes, such as cellulase, hemicellulase, and protease, are subsequently secreted and employed to enter the roots (Viterbo et al., 2004). *Trichoderma* spp. proliferation is further aided by the highly hydrated polysaccharides of the root-secreted mucigel layer, as well as the mono- and disaccharides discharged into the rhizosphere by plant roots. According to research, root colonization, defense mechanism coordination, and leaf photosynthetic rate enhancement are all facilitated by plant-derived sucrose. To obtain root exudates, *Trichoderma* spp. have a variety of transporters/carriers, such as permease/intracellular invertase system and a di/tripeptide transporter. Hydrophobins are tiny proteins secreted by *Trichoderma* spp. that have recently been discovered. These proteins aid *Trichoderma* spp. in the attachment of fungal roots. These proteins feature a unique domain with eight cysteine residues in conserved positions. Based on their hydropathy patterns and solubility, hydrophobins were initially classified as class I and II. Furthermore, phytopathogenic fungi also rely on them to attach to the surface of the host plant (Talbot et al., 1996). Recently, a gene *tvhydii1* that belong to class II hydrophobin, was isolated and characterized from *T. virens* (Guzmán-Guzmán et al., 2017). Overexpression of the gene *tvhydii1* increases *T. virens*’ antagonistic activity against *R. solani* as well as the colonization of plant roots. Furthermore, deletion of *tvhydii1* reduces antagonistic activity against *R. solani* and plant root colonization. *Trichoderma* spp. also encode expansin-like proteins, such as Swollenin TasSwo, which loosen, expand, or disrupt plant cell wall elements including cellulose and hemicellulose. Although their exact mechanism of action is unclear, it is thought that expansin-like proteins break into the crevices generated by interlacing microfibers in the cell wall, causing a conformational shift that causes the cell wall to expand, assisting root colonization. Plant CWDEs are also engaged in active root colonization, in addition to expansin and hydrophobin proteins. Many plants showed the strengthening of epidermal and cortical cell walls, as well as the deposition of considerable quantities of callose and cellulose, 72 h following root colonization. Callose-enriched cell walls limit *Trichoderma* spp. to the epidermis and cortical intercellular gaps, preventing *Trichoderma* spp. from entering the vascular stele. Antimicrobial chemicals are also synthesized and accumulated by plants in response to *Trichoderma* spp. invasion. To a large extent, the ability to colonize plant roots is determined by the strain’s ability to withstand environmental stresses. Several ATP-binding cassette (ABC) transporter genes have been discovered and described in *Trichoderma* spp., and they have been linked to the transfer of a variety of substrates, including phytotoxins, mating factors, antibiotics, pesticides, and heavy metals. The rapid degradation of phenolic compounds exuded by plants and the suppression of phytoalexin production, as detected in *Trichoderma* spp. is due to ABC transport systems (Cheng et al., 2011). ABC transport systems are key factors in the multiple interactions established by *Trichoderma* spp. with other microbes in a potentially toxic or antagonistic environment. *Trichoderma* spp. has been shown to contribute to biocontrol through small cysteine-rich proteins. These small proteins bind to chitin of plants and fungi and prevent *Trichoderma* spp. from chitinases (Stergiopoulos and de Wit, 2009).

## *Trichoderma* spp. Induced Resistance in Different Plants Against *Rhizoctonia solani*

### *Trichoderma*-Induced Systemic Resistance

**Figure 4:**
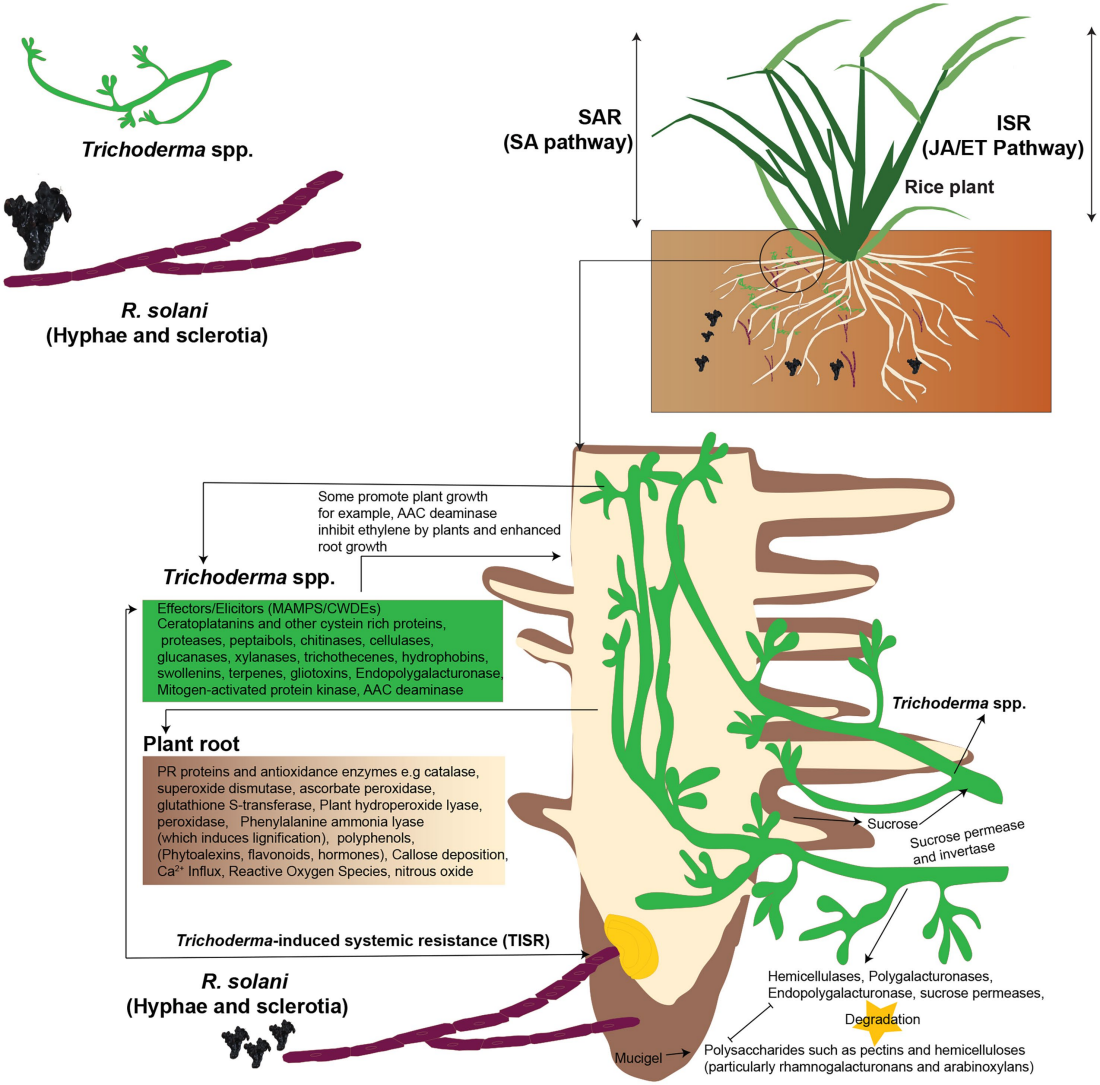
*Trichoderma*-induced systemic resistance (TISR) in plants against *R. solani*. Both SA and JA/ET-mediated signal transduction pathways may trigger defensive responses against *R. solani*, boosting plant resistance. *Trichoderma* spp. release enzymes to degrade plant polysaccharides, colonize the roots, and take sucrose as a carbon source by using sucrose permease and invertase enzymes. *Trichoderma* spp. produce elicitors, such as MAMPs to induce TISR in the plants; plants synthesize hydroperoxide lyase, peroxidase, and phenylalanine ammonia-lyase (which induces lignification) and deposit callose. *Trichoderma* MAMPs, such as xylanase, elicits plant defense responses against *R. solani*. The 1-aminocyclopropane-1-carboxylic acid (AAC) deaminase inhibits ethylene formation by the plant, and this leads to enhanced root growth, and this is due to the formation of hormones. Besides, *Trichoderma* spp. attach to plants roots by producing hydrophobins and swollenin. Reconstructed from Druzhinina et al. (2011).

Depending on the pathosystem, plant defense responses are usually triggered by activation of a complex signal transduction network that includes salicylic acid (SA), jasmonic acid (JA), either with or without ethylene (ET), and abscisic acid (ABA) as important plant immunity regulators. Checker et al. (2018), SA-mediated signaling pathways in plants result in systemic acquired resistance (SAR) against biotrophic and hemibiotrophic diseases. JA/ET-mediated signaling pathways, on the other hand, result in induced systemic resistance (ISR) in plants against necrotrophic diseases (Pieterse et al., 2014). According to a recent research, *Trichoderma* spp. induce a hybrid ISR/SAR type of resistance in plants against fungal pathogens including *R. solani* known as *Trichoderma*-induced systemic resistance (TISR; Leonetti et al., 2017; Figure 4). Hence, resistance in plants is increased by *Trichoderma* spp. against *R. solani*, which can be activated through signaling pathways that involve both SA and JA/ET-mediated signal transduction. Even still, scientists disagree on the method by which *Trichoderma* spp. activate defensive responses and the sort of resistance they induce in plants. Furthermore, there are significant gaps and differences when it comes to explaining the crosstalk of signaling molecules involved in *Trichoderma*-induced defense responses in plants. Other chemicals that may reduce or increase the synthesis and activity of signaling molecules must also be considered in order to better comprehend the crucial interactions between JA, SA, ET, and their derivatives in the intricate signaling network (Figure 4).

### Microorganism-Associated Molecular Patterns

Like fungi and mammals, plants activate possible defense systems in response to the presence of other species. This is best appreciated in the context of pathogens that induce a two-branched inborn immune response. PAMP-triggered immunity (PTI) is the first stage in which plants use pattern recognition receptors to recognize and respond to pathogen-associated molecular patterns (PAMPs) or microorganism-associated molecular patterns (MAMPs). Plants respond to pathogen virulence factors in the second step known as effector-triggered immunity (ETI). MAMPs are molecular signatures that are extremely conserved, such as fungi’s chitin and xylanase and oomycetes’ heptaglucan. After PRR activation, changes in ion fluxes across the plasma membrane, the oxidative burst (production of nitric oxide and reactive oxygen species), activation of MAPK cascades, and callose deposition are all downstream defensive activation events. Many breakthroughs have been made in identifying the pathways involved in this resistance; in many cases, SA or JA, when combined with ET, ROS, or NO, causes a cascade of processes that culminate in the synthesis of a range of metabolites and proteins with varied roles (Bellin et al., 2013). ABA, auxins, gibberellins, cytokinins, and brassinosteroids have also been demonstrated to play essential roles in recent studies (Pieterse et al., 2009). Although there appears to be crosstalk or competition between the pathways, different stresses stimulate distinct routes (Durrant and Dong, 2004). SAR confers long-term disease resistance by accumulating genes that produce pathogenesis-related (PR) proteins against pathogens that are either biotrophic or hemibiotrophic (Pieterse et al., 2009). However, rather than directly initiating PR gene transcription, ISR enhances plants’ defenses against a future onslaught by necrotrophic pathogen like *R. solani*. Overall, the JA/ET and SA pathways are thought to be mutually special. However, other investigations have found a synergistic effect between these routes. According to recent advances, *Trichoderma* spp. simultaneously induces plant SAR-related genes as well as ISR-related genes during plant root colonization, providing protection against *R. solani* with various lifestyles (Salas-Marina et al., 2011). Recent research has shown that *Trichoderma* spp. may cause biochemical and molecular alterations in SAR, which are mostly linked to the production of PR proteins, such as PR1, PR5, and PR2 (Zhang and Zhuang, 2020). The activation of the mutually antagonistic SA and JA pathways by *Trichoderma* spp. results in a loss of plant ecological fitness, a process known as crosstalk (Van Oosten et al., 2008). *Trichoderma*–plant interactions take place predominantly in the rhizosphere, where resistance development is driven by the interchange of microbial and plant elicitors required for organism-to-organism interactions. Depending on the involved elicitors created by *Trichoderma* hyphae, the interaction of these molecules with plant receptors may influence *Trichoderma* adhesion and identification, and hence the induction of resistance in plants. Secondary metabolites produced by *Trichoderma* spp., such as proteins with enzymatic activity, as well as *Trichoderma* spp. and plant cell wall components, are all implicated in the development of plant resistance. Pathogen defenses have been activated by plant components, such as pectins, phospholipids, and saccharides. Some *Trichoderma* elicitors, include proteins produced by avirulence genes and MAMPs, which are slow-evolving molecules. *Trichoderma* spp. secrete low molecular weights (6–42 kDa) enzymes or peptides which act as elicitors, such as serine xylanases, proteases, chitinases, cellulases, or glucanases. Other *Trichoderma* spp. compounds which act as elicitors are indole compounds, fatty acids, lipids and their derivatives (glycosphingolipids etc.), saccharides (polysaccharides or oligosaccharides) and chitin or chitin-like compounds (Djonović et al., 2007; da Silva Aires et al., 2012; Tables 4 and 5).

**Table 5:** Role of *Trichoderma* spp. genes which act as elicitors in resistance induction in different plants against *R. solani*.

Protein/molecule	Enzyme	*Gene*	Plant	*Trichoderma* spp.	References
Proteins	Endochitinase	*chit42*	Tobacco	*T. harzianum*	Lorito et al., 1998
	Endochitinase	*ThEn-42*	Tobacco	*T. harzianum*	Lorito et al., 1998
	Expressed sequence tags	*Epl1*	Rice, soybean	*T. asperellum*	Liu et al., 2010
	Endopolygalacturonase	*Thpg1*	Arabidopsis	*T. harzianum*	Morán-Diez et al., 2009
	Expressed sequence tags	*Epl1*	N.A.	*T. atroviride*	Seidl et al., 2006
	Mitogen-activated protein kinase	*tmkA*	Cucumber	*T. virens*	Viterbo et al., 2005
	Xylanase	*TasXyn29.4*	Popular	*T. asperellum*	Guo et al., 2021
	Xylanase	*TasXyn24.2*	Popular	*T. asperellum*	Guo et al., 2021
Chromatin remodeler	Helicase-related protein	*ipa-1*	Arabidopsis	*T. virens*	Estrada-Rivera et al., 2020
Cerato-platanins	Small extracellular cysteine-rich proteins	*Sm1* and *Ep11*	Cotton	*T. virens*, *T. atroviride*	Djonović et al., 2006
Transferase	4-Phosphopantetheinyl transferase	*ppt1*	Arabidopsis	*T. virens*	Velázquez-Robledo et al., 2011
Cellulase	Endoglucanases, exoglucanases, and β-glucosidases	*N.A*.	Tobacco, lima bean, corn cultures	*T. viride*	Aidemark et al., 2010
Protease	Aspartyl protease	*N.A*.	Cucumber	*T. virens*	Viterbo et al., 2004
Protease	Serine protease	*tvsp1*	Cotton	*T. virens*	Pozo et al., 2004
Chitinase	Endochitinase	*N.A*.	Cotton, rice		Kumar et al., 2009; Shah et al., 2009
Trichothecene	Trichodermin and harzianum A	*N.A*.	Tomato	*T. arundinaceum*	Malmierca et al., 2012
Class II hydrophobin family	Hydrophobin	*tvhydii1*	Arabidopsis	*T. virens*, *T. atroviride* and *T. reesei*	Guzmán-Guzmán et al., 2017

N.A., not available.

### Cerato-Platanins, Hydrophobins, Swollenins, and Expansins

Small proteins like cerato-platanins, hydrophobins, swollenins, and expansins are vital between *Trichoderma* spp. and plants. Host presence stimulates secretion of these proteins by *Trichoderma* spp. These proteins are also involved in mycoparasitism and induction of plants resistance against pathogens. For example, Cerato-platanins (*Sm1*/*Epl1*), which are tiny secreted proteins with four cysteines linked together by two disulfide linkages are necessary for *T. atroviride* and *T. virens* mediated cotton resistance to *R. solani*. They trigger systemic and local resistance in plants for *R. solani* (Djonović et al., 2006; Seidl et al., 2009). Hydrophobins are a group of other small proteins which are secreted from the *Trichoderma* spp. cell wall. They help *Trichoderma* spp. to adhere to the root surface. Local defense in plants is stimulated by Swollenin, expansin-like proteins with a cellulose-binding domain (Guzmán-Guzmán et al., 2017; Table 5). Swollenin disrupt plant cell walls’ crystalline cellulose structure and aid in *Trichoderma* spp. colonization of plants roots (Saloheimo et al., 2002). Root colonization by *T. asperellum* stimulates the production of swollenin to induce local defense in plant (Brotman et al., 2008). In the rhizosphere, *Trichoderma* spp. use swollenin to establish themselves by increase root surface area. Plant protein for expansion of root and root hair cell wall is expansins which has sequence similarity to Swollenin (Guo et al., 2011).

#### Xylanases

Glycosyl Hydrolase Family 11 bacteria secrete xylanases, which are essential CWDEs (GH11). These CWDEs have the ability to degrade xylan, a key component of plant cell walls. Plants can detect the loss of cell wall integrity when xylanases begin to breakdown the plant cell wall and trigger the defensive signaling system. Recently, xylanases (TasXyn29.4 and TasXyn24.2) were discovered as *Trichoderma* spp. MAMPs that induce plant defensive responses in Popular. The production of these *T. asperellum* xylanases in plants induced plant defense responses against *R. solani* attacks mediated by ethylene and H_2_O_2_ (Guo et al., 2021).

#### Chitinases

*Chit42*, a *T. harzianum* chitinase improved resistance of tobacco to *R. solani*. This show the importance of chitinases in the activation of resistance in plants (Lorito et al., 1998).

#### Proteases

*Trichoderma* spp. produce considerable amounts of proteases. *T. harzianum*, *T. asperellum*, and *T. virens* have aspartyl and serine proteases that are involved in both mycoparasitism and symbiotic relationship between *Trichoderma* spp. and plants, besides, enhancing defensive capability against *R. solani* (Viterbo et al., 2005). Additionally, these enzymes assist *Trichoderma* spp. in colonization of plants roots as well as the production of secondary metabolites such PR proteins and phytoalexins against *R. solani* (Viterbo et al., 2005).

#### Peptaibols and Trichothecenes

Another notable group of *Trichoderma* spp. secondary metabolites are peptaibols (peptaibiotic). The name peptaibols comes from a combination of the terms PEPTide, AIB, and alcohOLs, which are the distinguishing characteristics of peptaibols. Peptaibols are characterized by large quantities of non-standard amino acids (especially a-aminoisobutyric acid), 2-amino alcohol and a C-terminal 1. Peptaibols are not produced by ribosomes. Instead, multidomain enzymes on huge non-ribosomal peptide synthetase (NRPS) complexes produce them (Mukherjee et al., 2012). These peptaibols are also important elicitors produced mainly by *T. atroviride* and *T. virens* and are implicated in the development of pathogen resistance in plants (Mukherjee et al., 2012). Trichothecenes are also produced by *Trichoderma* spp., that is, *Harzianum A* and trichodermin which act as elicitors for plant resistance to *R. solani*. A tri4 gene mutation, for example, reduced antifungal action against *R. solani* as well as the ability to regulate the production of tomato plant defense-related genes from the SA and JA pathways (Malmierca et al., 2012). Furthermore, plant development and plant defense mechanisms are also induced by oxygen heterocyclic compounds (OHC), such as polyketides, harzianolides, harzianopyridone, and pyrones (esters), peptides (gliovirin and gliotoxin), non-polar chemicals (terpenoids and steroids), and anthraquinone pigments. However, plant resistance through *Trichoderma* spp. by reduction of VOCs, that is, ketones and aldehydes remain questionable.

## Role of MAMPs, Reactive Oxygen and Nitrogen Species, Transcription Factors, Defense-Related Genes and Enzymes

### *Trichoderma* spp. Release MAMPs for Molecular Recognition by Receptors of Plants

PRRs recognize MAMPs released by *Trichoderma* spp. By signaling molecules within the plants, these MAMPs contribute to the signal cascade. *Trichoderma* MAMPs transiently promote Ca^2+^ and H^+^ influx and K^+^ and Cl^−^ ejection in response to suitable plant receptors. Variations in the plasma membrane potential (Vm) occur often because of ion imbalances and changes in the channel activity (Liu et al., 2010). Plant cells undergo fast ROS, RNS and pH changes during depolarization. In addition to changes in ion absorption, *Trichoderma* spp. releases organic acids (gluconic or citric acid) into the soil, which lowers the soil pH. ROS and RNS are released during cell wall expansion and lignification. Furthermore, cell wall peroxidases and calcium channels become active. Many secondary metabolites and signaling molecules, including as H_2_O_2_ and NO, as well as SA and its derivatives, and JA/ET are generated and accumulated in response to *Trichoderma* spp. (Harman et al., 2004). *Trichoderma* spp. MAMPS and other effector molecules bind to PRRs and intracellular receptors in plants, triggering MTI (MAMPS-triggered) and ETI (effector-triggered) immunity. This interaction between *Trichoderma* spp. and plants produces ROS and RNS, which act as signaling molecules and initiate a defensive response in plants by synthesizing antifungal molecules, such as phytoalexins, VOCs (volatile organic compounds), PRs proteins, such as CWDEs, and so on. *Trichoderma* spp. has a local and systemic action that involves a signaling cascade and activation, as well as the accumulation of antimicrobial compounds and enzymes, such as polyphenol oxidase, peroxidase, lipoxygenase and PAL. PR proteins, terpenoids, phytoalexins (rishitin, phytosterol, lubimin, coumarin, resveratrol, solavetivone, and others), and antioxidants (glutathione, ascorbic acid, and others) are produced. Plants respond to fungal invasion by producing and concentrating defensive molecules, such as phytoalexins, aglycones, flavonoids, phenolic byproducts, terpenoids, and other antimicrobial substances. *Trichoderma* spp. on the other hand, are typically resistant to plant defense compounds and colonize plant root due to presence of ABC transport systems. Recent studies show that plants employ ROS and RNS as messengers to control the interplay between secondary messengers, MAPKs, and hormones, which are crucial in the induction of plant resistance SAR (Sami et al., 2018). An enzyme system known as the MAPKs kinase pathway may be activated by extracellular *Trichoderma* MAMPs, when they connect with plant receptors. The first step in triggering MPKKK kinase activity is a ligand–receptor contact. In the next step, MPK kinase is phosphorylated by MPKKKs, which in turn activates MPKK kinases, which phosphorylate MPKs (Sami et al., 2018).

### Reactive Oxygen and Nitrogen Species

In oxidative signaling, reactive oxygen (RO) and reactive nitrogen (RN) species interact with multiple hormonal signaling pathways. When plants were challenged with necrotrophic fungi, such as *R. solani*, among all RO and RN species, H_2_O_2_ and nitric oxide (NO) were shown to be quickly produced and were considered primary defensive activators. The processes by which NO may influence defensive signaling cascades were well investigated. S-nitrosylation and tyrosine-rich group nitration have emerged as significant NO-dependent protein regulatory mechanisms. Protein cysteine-rich thiol groups react with NO to create S-nitrosothiols so-called “reversible S-nitrosylation of proteins.” S-nitrosylation plays an important role in defensive responses, as shown by its effects on the SA signaling protein NPR1 and the ROS-generating NADPH oxidase complex AtRBOHD. NO has been shown to work with ROS and SA to establish SAR in plants (Sami et al., 2018). NO is also linked to other resistance-inducing signaling pathways, such as JA and ET. NO *via* S-nitrosylation has been shown in recent research to be one of the key regulators of SA-dependent systemic defensive responses in *T. atroviride*-treated plants. SA play an important part in the systemic defense responses elicited by *T. atroviride* in cucumber plants, protecting them from *R. solani*. SA or increasing the quantity of H_2_O_2_ or NO could promote the synthesis of active NPR1 proteins, which regulate the expression of genes that code for plant defense proteins. NPR1, which resides as an oligomer in the cytoplasm and is held together by intermolecular disulfide bonds, is susceptible to changes in redox status. By modifying the state of cell reduction, accumulated SA, H_2_O_2_ and NO might diminish disulfide bonds, leading NPR1 to degrade into monomers, which, when transferred into a nucleus to induce TISR by activating pathogenesis-related (PR) genes (Nawrocka et al., 2019). The mitogenic kinase pathways are also activated, resulting in the activation of transcription factors against *R. solani* (Nawrocka et al., 2019).

### Transcription Factors, Defense-Related Genes, and Enzymes

#### Transcription Factors and Pathogenesis-Related Genes

Transcription factors modulate the expression of certain genes required for many key physiological activities and stress responses, acting as regulators of gene transcription. The *WRKY* transcription factor family has been connected to abiotic stress, growth, and development in addition to plant–microbe interactions. As discussed before, SAR often results in increased levels of SA and coordinated activation of PR genes, such as *PR5*, *PR2*, and *PR1* involving one or more signaling molecules, that transmit an elevated immune response against *R. solani*. For example, when bean plants were exposed to *T. velutinum* in the absence of *R. solani*, *WRKY33* gene expression increased considerably whereas *PR1* expression decreased. However, when beans plants were just exposed to *R. solani*, *WRKY33* gene expression was reduced whereas *PR1* gene expression remained unaffected. Furthermore, when bean plants were exposed to *T. velutinum* and *R. solani*, the genes *WRKY33* and *PR1* were both downregulated (Mayo et al., 2016). Furthermore, *T. asperelloides* colonization of *A. thaliana* roots triggered a rapid increase in expression of *WRKY* transcription factors, which suppressed SA signaling and triggered JA pathway responses against *R. solani*. These findings imply that WRKY proteins, which are well-known *PR* gene activators, play a key role in chromatin modifications that enhance gene expression (Mayo et al., 2016). PR gene expression, including enzymes, such as cellulases, glucanases, and chitinases, is engaged in direct control of *R. solani* and plant biochemical barrier reinforcement (Heflish et al., 2021). In another study, the interaction of bean plants with *R. solani* resulted in the downregulation of seven defense-related genes, including chitinases (*CH5b*, *CH1*), *PR1*, *PR2*, *PR3*, *PR4*, and *PAL*, as a mechanism to overcome the plant defense response, allowing the infection process to progress within the plant. Ergosterol is a sterol present in the fungal membrane that, although being classified as a MAMP by the plant, causes a sequence of events that result in the activation of defense-related genes. Squalene (polyunsaturated terpene) is a precursor in the biosynthesis of ergosterol. In one study, higher ergosterol and squalene synthesis by *Trichoderma* spp. resulted in the activation of defense-related genes in bean plants against *R. solani* (Mayo et al., 2015).

#### Defense-Related Genes and Enzymes

Many studies have shown that plants treated with *Trichoderma* spp. boosted the activity of defense-related enzymes, such as peroxidase, chitinase, peroxidase, -1, 3-glucanase, phenylpropanoids, polyphenol oxidase, superoxide dismutase, chitinase, and phenylalanine ammonia-lyase (PAL). When cotton seeds were treated with *T. virens*, for example, peroxidase activity was increased in the roots of treated cotton plants (Howell, 2003). Furthermore, by boosting ROS scavenging enzymes, *Trichoderma* spp. contribute to plant resistance to *R. solani*. *Trichoderma harzianum*, for example, increases the activities of ascorbate peroxidase (APX), guaiacol peroxidase (GPX), superoxide dismutase (SOD), and catalase (CAT) in tomato plants against *R. solani* (Youssef et al., 2016). *Trichoderma* spp. also produce lytic enzymes, such as chitinase and β-1,3-glucanase, which break down *R. solani* chitin and β-1,3-glucan components. During an *R. solani* attack, for example, a bean chitinase promoter is substantially activated in transgenic tobacco plants (Roby et al., 1990). The protection against *R. solani* given by the expression of certain chitinase genes from *Trichoderma* spp. or other plants is remarkable. Defense-related gene expression and chitinase enzyme activity, for example, confer high resistance to *R. solani* infection in transgenic cotton plants expressing an endochitinase gene from *T. virens* (Kumar et al., 2009). Cellulases from *Trichoderma* spp. have also been shown to produce ISR in plants *via* the ET or JA pathways. Furthermore, *T. viride* and *T. harzianum* with biocontrol capacity to protect rice plants against *R. solani* exhibited negative morphological and physiological changes in the pathogen hyphae, such as swelling, knotting, crumpling, flattening, shriveling, bursting, and cytoplasm leakage. Furthermore, the increase of defense-related enzymes has been noted (Singh et al., 2016). *Trichoderma virens* promoted ISR in tomato plants by activating defense enzymes, such as GPX, syringaldazine peroxidase (SPX), and PAL against *R. solani* in another study. As a result, the buildup of secondary metabolites, such as phenols and H_2_O_2_, was increased, but lipid peroxidation was reduced in the leaves (Małolepsza et al., 2017). As a result, *Trichoderma* spp. treatment of plants produced disease resistance against *R. solani* by reprogramming the pathways and cascades involved in several defense-related activities (Zeilinger and Atanasova, 2020). The conclusion is that TISR is a complex occurrence, and the current findings do not reflect a thorough grasp of the processes and reactions to *R. solani.*

## Conclusion and Future Perspectives

To date, the best biocontrol agents described against *R. solani* are *Trichoderma* spp. Antagonism of *R. solani* is linked to a variety of *Trichoderma* spp. genes. As previously mentioned, antagonism is dependent on a number of genes for sensing, signaling, antibiosis, and mycoparasitism. In addition, many *Trichoderma* spp. genes are involved in competition and systemic resistance induction in plants for *R. solani*. In Figures 2–4, we described the *Trichoderma* spp. genes involved in antagonism of *R. solani*, what happens to *R. solani* when it is parasitized by *Trichoderma* spp. and how *Trichoderma* spp. induce resistance against *R. solani*. As many *Trichoderma* spp. genes are involved in the antagonism of *R. solani*, making the molecular mechanisms underlying the antagonistic effects more complicated. Hence, to completely comprehend the effect of *Trichoderma* spp. genes against *R solani*, additional studies are required. In conclusion, *Trichoderma* spp. possess a diverse set of genes that produce secondary compounds which can parasitize and antagonize *R. solani*. Systemic resistance against *R. solani* by *Trichoderma* spp. largely because of variety of metabolites produced against it. *Trichoderma* spp. also have a diverse set of effectors and elicitors recognized by plant receptors to activate signaling and gene regulation, which serves as the foundation for *Trichoderma* spp. to develop *R. solani* defense responses, as shown in Table 5. More research into the molecular, physiological, and biochemical underpinnings of *Trichoderma* spp. activity as multifunctional biocontrol agents is required to fully comprehend the impact of *Trichoderma* spp. on plants and their practical utility in plant protection against *R. solani*. The chemical nature of a number of secondary metabolites generated by *Trichoderma* spp. against *R. solani* is still unknown. Furthermore, there is a need to understand the molecular communication between *Trichoderma* spp. and plants in the presence of *R. solani*. Due to their precision, sensitivity, and specificity, efficient and sophisticated Next-generation sequencing technologies are currently used in studies involving *Trichoderma* spp. and *R. solani*. They will hopefully fill a gap in *Trichoderma* spp. biocontrol studies against *R. solani* when combined with other methods, such as metabolomics, metagenomics, proteomics, and bioinformatics.

## Author Contributions

AA and MM: writing-original draft and figure preparations. MAS, QS, MKS, SS, BKK, MCZR, SH, and EAM-E: collecting literatures, software, tables preparations, and editing. HZ, YI, and LZ: supervision, project administration, resources, and funding acquisition. All authors have read and agreed to the published version of the manuscript. All authors listed have made a substantial, direct and intellectual contribution to the work and approved it for publication.

## Funding

This work was supported by the China Postdoctoral Science Foundation (no: 2014M561669) and the Zhejiang Academy of Agricultural Sciences “high-talent introduction and ongoing training fund” (grant no: 10300000021LL05).

## Conflict of Interest

The authors declare that the research was conducted in the absence of any commercial or financial relationships that could be construed as a potential conflict of interest.

## Publisher’s Note

All claims expressed in this article are solely those of the authors and do not necessarily represent those of their affiliated organizations, or those of the publisher, the editors and the reviewers. Any product that may be evaluated in this article, or claim that may be made by its manufacturer, is not guaranteed or endorsed by the publisher.
